# Clinical and experimental aspects of aneurysmal subarachnoid hemorrhage

**DOI:** 10.1111/cns.13222

**Published:** 2019-10-03

**Authors:** Badih J. Daou, Sravanthi Koduri, B. Gregory Thompson, Neeraj Chaudhary, Aditya S. Pandey

**Affiliations:** ^1^ Department of Neurological Surgery University of Michigan Ann Arbor Michigan

**Keywords:** aneurysm, subarachnoid hemorrhage, vasospasm

## Abstract

Aneurysmal subarachnoid hemorrhage (aSAH) continues to be associated with significant morbidity and mortality despite advances in care and aneurysm treatment strategies. Cerebral vasospasm continues to be a major source of clinical worsening in patients. We intended to review the clinical and experimental aspects of aSAH and identify strategies that are being evaluated for the treatment of vasospasm. A literature review on aSAH and cerebral vasospasm was performed. Available treatments for aSAH continue to expand as research continues to identify new therapeutic targets. Oral nimodipine is the primary medication used in practice given its neuroprotective properties. Transluminal balloon angioplasty is widely utilized in patients with symptomatic vasospasm and ischemia. Prophylactic “triple‐H” therapy, clazosentan, and intraarterial papaverine have fallen out of practice. Trials have not shown strong evidence supporting magnesium or statins. Other calcium channel blockers, milrinone, tirilazad, fasudil, cilostazol, albumin, eicosapentaenoic acid, erythropoietin, corticosteroids, minocycline, deferoxamine, intrathecal thrombolytics, need to be further investigated. Many of the current experimental drugs may have significant roles in the treatment algorithm, and further clinical trials are needed. There is growing evidence supporting that early brain injury in aSAH may lead to significant morbidity and mortality, and this needs to be explored further.

## 1 INTRODUCTION

Subarachnoid hemorrhage (SAH) has a reported incidence between 10 and 15 people per 100 000 in the United States.^1^, ^2^ Despite significant improvements in treatment modalities and critical care management, SAH is associated with significant morbidity, mortality, and socioeconomic impact. This is not only as a result of the hemorrhagic event, but further related to the sequelae of the irritating blood by‐products that can lead to cerebral vasospasm and delayed cerebral ischemia (DCI) and can impair the circulation of cerebrospinal fluid (CSF) with resultant hydrocephalus. The absence of prevention strategies or efficacious therapy of cerebral vasospasm and DCI has resulted in rigorous research efforts. A multitude of animal models have been designed to address the complex pathogenesis of cerebral vasospasm. The preclinical animal studies have led to multiple clinical trials evaluating different potential therapeutic strategies. Available treatments for aneurysmal SAH continue to expand as research continues to identify new therapeutic targets. We intended to review the clinical and experimental aspects of aSAH and identify strategies that are being evaluated for the treatment of cerebral vasospasm.

## 2 METHODS

A literature review on aSAH was performed using PubMed. The following search terms were used: aneurysmal subarachnoid hemorrhage (aSAH), cerebral vasospasm, DCI, hydrocephalus, triple‐H therapy, nimodipine, magnesium, milrinone, statins, endothelin receptor antagonists, tirilazad, fasudil, cilostazol, albumin, corticosteroids, minocycline, deferoxamine, intrathecal therapy, intraventricular therapy, intraaterial therapy, angioplasty, and animal models of SAH. We evaluated studies in English that investigated interventions for managing aSAH.

## 3 RESULTS

### 3.1 Presentation and diagnosis of SAH

Most SAHs are caused by a ruptured intracranial aneurysm and are referred to as aSAH. The prevalence of intracranial aneurysms is about 3%.^3^

The primary symptom of aSAH is a sudden, severe headache classically described as the "worst headache of life," and may be accompanied by a brief loss of consciousness, nausea or vomiting, meningismus, and seizures.^4^ At least 10%‐15% of patients with aSAH die before reaching the hospital. Noncontrast head computed tomography (CT) scan is the mainstay of diagnosis of SAH.^5^ Cerebral angiography is then pursued to identify the etiology of SAH. Both CT angiography (CTA) and magnetic resonance angiography (MRA) can identify aneurysms 3‐5 mm or larger with a high degree of sensitivity,^6^ but digital subtraction cerebral angiography remains the gold standard test to identify the etiology of SAH. Lumbar puncture can be considered if there is a strong suspicion of SAH despite a normal head CT. A patient presenting with aSAH is admitted to an intensive care unit for hemodynamic and neurological monitoring, and commonly ventriculostomy placement for drainage of CSF and recording of intracranial pressure. This is followed by definitive treatment of the ruptured aneurysm or pathology causing the SAH.^5^

### 3.2 Treatment of cerebral aneurysms

Treatment of cerebral aneurysms falls primarily under two treatment categories: surgical clipping and endovascular treatment.^7^, ^8^ Surgical management of cerebral aneurysms typically consists of clip placement across the neck of the aneurysm. It has been proven to be an effective and safe procedure and was long the most practiced technique for treatment of intracranial aneurysms. Endovascular therapy for cerebral aneurysms was introduced in the early 1990s with the advent of the Guglielmi platinum detachable coils.^9^ Access is typically obtained through the femoral artery, and with the assistance of catheters and wires, the aneurysm is reached and coils are deployed. A local thrombus then forms around the coils, obliterating the aneurysmal sac. More recent techniques have evolved to target complex aneurysms. These include stent‐assisted coiling,^10^ balloon‐assisted coiling,^11^ flow diverters,^12^ and embolic agents.^13^

Multiple factors affect the approach utilized to treat a cerebral aneurysm, most notably aneurysm location, size, and neck size. Endovascular treatment is often the preferred technique for posterior circulation aneurysms and ruptured aneurysms in elderly patients (>70 years of age) given higher surgical risks.^14^ In contrast, aneurysms in the middle cerebral artery, ruptured aneurysms with large intraparenchymal hematomas, are most commonly approached surgically.^5^ Combined endovascular and surgical techniques may be required with some very large or complex aneurysms.

The ISAT, a multicenter randomized trial comparing microsurgical and endovascular treatment, randomized 2143 patients with aSAH.^7^ One‐year outcomes demonstrated a reduction in death and disability from 31% in the microsurgery group to 24% in the endovascular arm (relative risk reduction of 24%). The risk of epilepsy and significant cognitive decline was also reduced in the endovascular group. Rebleeding occurred more commonly in the endovascular treatment group (2.9% after endovascular repair vs 0.9% after open surgery). The rate of complete aneurysm occlusion was lower with coiling (58% compared with 81% of clipped aneurysms). Due to lower morbidity, endovascular approaches are gaining more and more popularity. Both microsurgical and endovascular approaches are evaluated on a case‐to‐case basis.

A multitude of secondary events may arise after aneurysmal rupture, some even after securing the aneurysm, and may contribute to brain injury and affect clinical outcomes. (Table 1).

**Table 1:** Events/complications encountered in patients with aSAH after presentation and on follow‐up

Early	Late	Chronic
Rebleeding	Cerebral vasospasm	Chronic hydrocephalus that may require placement of a shunt
Acute hydrocephalus	Delayed cerebral ischemia	Behavior, personality, and memory changes
Seizures	Pituitary dysfunction	Need for further retreatment for recurrent or residual aneurysm
Cardio‐pulmonary issues and stress‐induced cardiomyopathy	Infection related to surgery or ventriculostomy.	
Sodium and electrolyte abnormalities		

Abbreviation: aSAH, aneurysmal subarachnoid hemorrhage.

### 3.3 Hydrocephalus

Hydrocephalus after aSAH is thought to be caused by obstruction of CSF flow by blood products or adhesions, or by a reduction of CSF absorption at the arachnoid granulations, or by an increase in CSF secretion.^15^ The reported rate of hydrocephalus after aSAH ranges from 15% to 87% on CT imaging.^5^ Acute hydrocephalus associated with aSAH is usually managed by external ventricular drainage or lumbar drainage. Acute CSF diversion for patients who develop hydrocephalus is shown to improve neurological outcomes.^16^, ^17^, ^18^

Chronic hydrocephalus associated with aSAH is usually treated with a shunt placement for continuous CSF drainage, typically a ventriculoperitoneal shunt.^19^ Not all patients with aSAH‐associated acute hydrocephalus develop shunt‐dependent chronic hydrocephalus. Besides CSF diversion, there are no other medical or surgical interventions that are proven to reduce the rate of post‐aSAH hydrocephalus.

### 3.4 Rebleeding

Treatment of the ruptured aneurysm should be performed as early as possible to reduce the rate of rebleeding after aSAH. Rebleeding occurs in 7%‐23% of patients and is a major predictor of poor outcome in patients with aSAH.^20^, ^21^, ^22^ Most studies have found that the risk of rebleeding is highest in the first 24 hours after aSAH.

Patients are at substantial risk of rebleeding early after presentation if the aneurysm is unsecured, with reported rates of rebleeding ranging from 4% to 13.6% within the first 24 hours. The risk is at its highest within six hours of the initial hemorrhage. The risk of rebleeding drops later to 1%‐2% each day in the first month following the hemorrhage.^5^, ^21^, ^23^, ^24^

The mortality associated with rebleeding is reported to be as high as 70%.^25^ The main factors that increase the risk of rebleeding are longer time to aneurysm treatment, worse neurological condition on presentation, larger aneurysm size, and persistently elevated systolic blood pressure.^21^, ^24^ Hypertension should be controlled after aSAH and until aneurysm treatment. A decrease in systolic blood pressure to <160 mm Hg is reasonable.^5^

Antifibrinolytic therapy has been shown to reduce the incidence of aneurysm rebleeding when there is a delay in aneurysm obliteration. This may increase the risk of deep venous thrombosis.^26^ For patients with an unavoidable delay in obliteration of aneurysm, and no contraindications, short‐term (<72 hours) therapy with tranexamic acid or aminocaproic acid could be attempted to decrease the risk of early aneurysm rebleeding.^5^

### 3.5 Vasospasm

Cerebral vasospasm after aSAH is defined as narrowing of the large and medium‐sized intracranial arteries. It represents one of the major causes of morbidity and mortality in patients with aSAH who reach the hospital and are undergoing medical care. The risk of vasospasm typically begins around day 3 and peaks around days 7‐10. The risk typically resolves 21 days after the bleed.^5^ Angiographic vasospasm is seen in 30%‐70% of patients with aSAH, and clinical or symptomatic vasospasm is seen in 20%‐30% of patients.^27^, ^28^ Cerebral vasospasm is a clinical diagnosis, and radiographic studies are utilized to support the diagnosis. The gold standard for detecting cerebral vasospasm remains digital subtraction angiography, and it has the advantage of being both a diagnostic and therapeutic tool. CTA is often employed as well for the detection of cerebral vasospasm, as it can provide a quick tool for diagnosis and monitoring of vasospasm. CT perfusion is another tool that can be useful to assess for cerebral ischemia and infarction as a result of vasospasm.^29^ Transcranial Doppler sonography (TCD) is widely used for detecting and monitoring vasospasm in aSAH in the clinical setting. It is often used daily in the first 2 weeks after aSAH and can follow the temporal course of vasospasm. TCD is highly operator‐dependent, and data obtained from TCD should always be correlated with how the patient is doing clinically.^30^

Cerebral vasospasm can lead to regional cerebral hypoperfusion and DCI, which constitutes a major cause of death and disability in patients with aSAH. DCI typically causes neurological deterioration or new focal neurological deficits.

Vasospasm leads to DCI in approximately 20%‐30% of patients with aSAH.^31^, ^32^, ^33^

Though the exact mechanism of how vasospasm occurs is still not completely elucidated, there are many proposed mechanisms, with cerebral vasospasm likely being a multifactorial pathology. The major proposed pathways contributing to vasospasm include the following: endothelial damage and formation of microthrombi, smooth muscle contraction from lysis of subarachnoid blood clots and blood degradation products and hemoglobin released into the subarachnoid space, decreased nitric oxide production leading to prolonged vasoconstriction, increased production and release of the potent vasoconstrictor endothelin‐1, cortical spreading depolarization, inflammation‐mediated oxidative stress and free radical damage to smooth muscle cells, and upregulation of apoptosis pathways following aSAH (Table 2).^34^, ^35^, ^36^, ^37^, ^38^, ^39^

**Table 2:** Proposed mechanisms underlying cerebral vasospasm and DCI

Prolonged smooth muscle contraction from lysis of subarachnoid blood clots and blood degradation products
Endothelial damage and formation of microthrombosis
Decreased nitric oxide production leading to prolonged vasoconstriction
Increased production and release of the potent vasoconstrictor endothelin‐1
Cortical spreading depolarization increasing metabolic demand and decreasing blood supply
Inflammation‐mediated oxidative stress and free radical damage to smooth muscle cells
Upregulation of apoptosis pathways following aSAH

Abbreviations: aSAH, aneurysmal subarachnoid haemorrhage; DCI, delayed cerebral ischemia.

As a consequence of the significant neurological morbidity or mortality of cerebral vasospasm and DCI, research has heavily focused on understanding the physiological mechanisms behind these events and developing effective preventive and therapeutic measures (Table 3). The cornerstone of vasospasm treatment continues to be focused on prevention given risk for worse clinical outcomes in patients who develop this condition.

**Table 3:** Agents and interventions targeting vasospasm in aneurysmal subarachnoid hemorrhage

	Mechanism of action in vasospasm	Use in clinical practice	Comment
Hyperdynamic and “triple‐H” therapy	Increase the mean arterial pressure and increase cerebral perfusion	Hypervolemia and hemodilution have fallen out of practice. Induced hypertension is utilized to reduce the risk of ischemia in patients who develop vasospasm but is not used as a prophylactic measure for all patients.	Systematic reviews concluded that “triple‐H” therapy does not appear to be beneficial for the prevention of vasospasm. Hypervolemia may increase the risk pulmonary edema, myocardial ischemia, and cerebral edema; maintaining euvolemia is recommended.
Nimodipine	Calcium channel blocker causing vasodilatation of vascular smooth muscle cells. Neuroprotective effect could be related to resistance to calcium‐mediated excitotoxicity and a reduction in the formation of microthromboses	Oral nimodipine should be initiated as soon as possible after aSAH at a dose of 60 mg every 4 h and maintained for about 21 d following SAH	No convincing evidence that nimodipine affects the incidence of either angiographic or symptomatic vasospasm But, nimodipine has been shown to improve neurological outcomes and to decrease mortality in patients with aSAH and is part of the standard of care due to its neuroprotective role.
Nicardipine, nitroprusside, and verapamil	Other calcium channel blockers causing vasodilation of vascular smooth muscle cells.	Frequently utilized for blood pressure management in aSAH, especially nicardipine, but not routinely tailored for vasospasm prophylaxis and treatment	May reduce vasospasm but indeterminate effects on clinical outcomes. Benefit does not surpass that of nimodipine
Magnesium	Vasodilatation by blocking voltage‐dependent calcium channels. Magnesium may also block the release of glutamate, providing a potential neuroprotective benefit	Not routinely used in all patients with aSAH and has fallen out of standard practice given inconsistent reports on its benefits. Correcting hypomagnesaemia is highly recommended.	Early animal studies and a phase II trial (MASH) reported positive results with intravenous magnesium. IMASH and MASH‐2, two phase III trials, did not show significant clinical benefit
Milrinone	Phosphodiesterase 3 inhibitor that leads to an increased level of intracellular cAMP causing vasodilatation. Potential antiinflammatory effects at the cerebral vessel wall.	Currently used in clinical practice occasionally in cases of refractory vasospasm not responsive to other measures and utilized intraarterially in patients who undergo endovascular interventions for symptomatic vasospasm.	Studies on intravenous and intraarterial milrinone have shown a safe profile and promising results in improving vasospasm. Overall role in improving patient outcomes still needs to be evaluated.
Statins	Improve cerebral vasomotor reactivity by upregulating endothelial nitric oxide synthase and increasing nitric oxide biosynthesis. May play a role in decreasing glutamate‐mediated excitotoxicity and help control the inflammatory response	Given the relative safety of statins, they are still used in clinical practice (pravastatin 40 mg daily or simvastatin 80 mg daily). The latest AHA/ASA recommendations state that it is reasonable to administer statin therapy to patients after aSAH to prevent vasospasm despite lack of strong evidence of benefit. Therapy should be started early (within 48 h) and continued for 14‐21 d.	Positive early trial showing benefit on decreasing the incidence of vasospasm, but more recent studies have not reported significant findings including the STASH phase III trial
Endothelin receptor antagonists (clazosentan, TAK‐044)	Inhibit the binding of endothelin 1, a vasoconstrictive peptide that is overproduced in SAH	Not used in common practice due to prominent side effects (pulmonary edema, hypotension, cerebral infarction anemia, and hypotension)	Endothelin receptor antagonists may have a role in reducing vasospasm especially at a high dose with inconsistent effect on functional outcome and important side effects
Tirilazad	Free radical scavenger and antioxidant effects, likely neuroprotective role	Not currently used in standard practice	Studies have not proven a consistent clinical benefit. A meta‐analysis involving 3821 patients found no significant difference with tirilazad compared with placebo with regard to death or poor outcome; fewer patients, however, developed DCI in the tirilazad group.
Fasudil	Rho‐kinase inhibitor that acts a vasodilator	Fasudil is currently utilized in Japan for vasospasm prophylaxis. Because of the limited number of trials, it is not as widely endorsed elsewhere.	Several trials reported a reduced rate of angiographic and symptomatic vasospasm improved clinical outcomes. Large randomized, controlled clinical trials are needed to further establish the benefit.
Cilostazol	Platelet aggregation inhibitor may have a role in preventing microthrombi formation and has a vasodilatory effect by inhibiting phosphodiesterase 3 and increasing intracellular cAMP	Not used routinely	Cilostazol seems to be a safe and promising agent. One meta‐analysis showed a decreased risk of symptomatic vasospasm, cerebral infarction, and poor outcome. Further large multicenter trials are needed.
Albumin	Neuroprotective properties likely related to its antioxidant and antiinflammatory properties. It increases oncotic pressure and reduces cerebral edema, increases neuronal survival, and maintains blood‐brain barrier integrity.	Used occasionally for volume resuscitation in SAH. Not routinely used for vasospasm prophylaxis or treatment.	ALISAH study identified that 1.25 g/kg/d of albumin is safe in patients with aSAH and may be associated with improved outcomes with no major complications. Further studies are currently underway.
Eicosapentaenoic acid	Inhibits Rho‐kinase activation and smooth muscle contraction.	Not currently used in the clinical setting	Early reports showed positive results, and further studies are needed.
Heparin and low molecular weight heparin	Attenuates inflammatory response, this is different than its function in anticoagulation.	High‐dose heparin/low molecular weight heparin infusions are not currently recommended routinely given concern for hemorrhage. Low doses are used for prophylaxis against venous thromboembolism	Inconsistent results with concern for hemorrhage.
Erythropoietin	neuroprotective properties through an indeterminate mechanism that seems to be unrelated to its function in erythropoiesis	Not commonly used in practice	The overall mechanisms and role in vasospasm are still unclear as well as the effect on clinical outcomes.
Corticosteroids	Antiinflammatory properties	Not commonly used in practice	A randomized trial that administered high‐dose methylprednisolone (16 mg/kg) reported better functional outcome at one year. Further studies are needed.
Minocycline	Antiinflammatory effects as well as serving as an iron chelator. Likely neuroprotective role.	Not commonly used in practice	Multiple trials have evaluated minocycline in acute ischemic stroke and intracranial hemorrhage patients with promising results and with a good safety profile. Role currently being evaluated in SAH.
Deferoxamine	Iron chelator. May target iron and hemoglobin‐induced early brain injury	Not commonly used in practice	Could be a promising agent; currently being evaluated in ICH and SAH and has reached clinical trials in ICH.
Intrathecal thrombolytics	Clearance of blood from the basal cisterns and ventricular system.	Not commonly used in practice	A meta‐analysis showed an absolute risk reduction in DCI, poor clinical outcome, and mortality. Intrathecal thrombolytics are still being investigated and not currently recommended.
Transluminal balloon angioplasty	Disruption of smooth muscle cells or extracellular matrix with resultant dilatation of the vessel and increased cerebral blood flow	Commonly used in patients with symptomatic vasospasm not responding to other medical measures. Not recommended for vasospasm prophylaxis.	Studies support this tool in the treatment of symptomatic vasospasm with improvement in vasospasm and patient outcomes. Large affirmative trials are missing, however.
Intraarterial papaverine	Increases cyclic AMP and causes vasodilatation	Not routinely used	There are significant limitations with its use and prominent side effects. Its use is not recommended in treating vasospasm.
Intraarterial verapamil, nicardipine, nimodipine, and milrinone	Calcium channel blockers causing vasodilatation of vascular smooth muscle cells.	Commonly used in patients with symptomatic vasospasm especially when involving small distal vessels or as adjunct to transluminal balloon angioplasty	Randomized trials are still needed to establish effects on clinical outcomes and to compare the different intraarterial treatments.

Abbreviation: aSAH, aneurysmal subarachnoid hemorrhage.

### 3.6 Therapies investigated in managing vasospasm

#### 3.6.1 Hyperdynamic therapy

Hyperdynamic therapy, including induced hypertension, hypervolemia, and hemodilution, referred to as "triple‐H" therapy, has been used as a method to prevent and treat cerebral vasospasm.^40^, ^41^, ^42^ The goal of this technique is to raise the mean arterial pressure and increase cerebral perfusion. It was one of the mainstays of vasospasm management and prevention. However, studies failed to show benefit of prophylactic triple‐H therapy and highlighted some notable risks.^41^, ^42^, ^43^ This strategy has fallen out of favor and is not recommended any longer.

Studies have shown that hypervolemic therapy did not prevent DCI when compared to euvolemia; in fact, hypervolemia may increase the risk pulmonary edema, myocardial ischemia, and cerebral edema.^44^ In a randomized trial, prophylactic hypervolemia caused an overall fourfold increase in the risk of adverse events.^45^ The current guidelines have shifted toward a more euvolemic approach with main goal of avoiding hypovolemia without volume overload; prophylactic hypervolemia before the development of angiographic vasospasm is not recommended.^5^

Hemodilution is thought to increase global CBF by decreasing blood viscosity. However, it results in reduced oxygen delivery capacity and may not increase the overall cerebral oxygen delivery.^46^ It has mostly fallen out of favor as a current treatment modality for vasospasm. In spite of widespread early adoption, primarily based on retrospective studies, systematic reviews of “triple‐H” therapy for prophylaxis of vasospasm concluded that it does not appear to be beneficial for the prevention of vasospasm.^42^, ^47^

Some components of this regimen are still currently in practice for treatment of symptomatic vasospasm, but not typically employed as a prophylactic measure for all patients. The induced hypertension component of “triple‐H” therapy is more effective in increasing CBF than hypervolemia and hemodilution. The loss of innate autoregulation during vasospasm makes cerebral perfusion pressure even more dependent on systemic blood pressure.^48^ This strategy is not used a preventive tool, but aims to reduce the risk of ischemia in patients developing vasospasm. Induced hypertension increases cerebral blood flow and brain tissue oxygenation, and may help in reversing neurological deficits. Induced hypertension relies on use of pressors, notably norepinephrine, dopamine, and phenylephrine, all of which have been shown to be successful in improving CBF and neurological outcome.^49^

#### 3.6.2 Nimodipine

Nimodipine is a calcium channel blocker originally used for blood pressure management. It crosses the blood‐brain barrier and causes vasodilation of vascular smooth muscle cells and thus is a main tool used in aSAH.

Nimodipine was initially studied in patients with aSAH as a means to prevent vasospasm. However, there is no convincing evidence that nimodipine affects the incidence of either angiographic or symptomatic vasospasm.^50^ But, nimodipine has been shown to improve neurological outcomes and to decrease mortality in patients with aSAH and is part of the standard of care due to its neuroprotective role.^51^, ^52^

Per the AHA/ASA recommendations, oral nimodipine should be initiated as soon as possible, typically at a dose of 60 mg every 4 hours, and maintained for about 21 days following SAH.^5^ The underlying mechanism for these effects is unclear, but it is known to be separated from its vasodilatory effects and could be related to resistance to calcium‐mediated excitotoxicity and a reduction in the formation of microthromboses, which play a role in development of vasospasm and DCI.^36^

Nimodipine is the only FDA‐approved drug to improve outcomes in aSAH. It has been reported to reduce the incidence of poor neurological outcome by 40%.^53^ In a trial by Allen et al^51^ that included 125 SAH patients, 1 of 56 patients who received nimodipine suffered from neurological deficit or death, which was statistically significant when compared to patients given a placebo (8 of 60 patients, *P* = .03). Several additional trials have confirmed the benefit of nimodipine in reducing the incidence of DCI and improving outcomes in SAH. A meta‐analysis that analyzed the results of 16 trials evaluating the role of calcium channel blockers in aSAH, encompassing 3361 patients, concluded that calcium channel blockers reduce the risk of poor outcome and secondary ischemia after aneurysmal SAH with oral nimodipine producing the most significant benefit and only with modest risks, systemic hypotension being the most common side effect of nimodipine.^52^

Other calcium channel blockers have been studied for treatment of vasospasm and DCI, most notably nicardipine, nitroprusside, and verapamil. There may be a role for the other calcium channel blockers, but the benefit does not surpass that of nimodipine.^52^ In a trial on nicardipine that treated 449 patients with IV nicardipine vs 457 patients with placebo, nicardipine‐treated patients had significantly reduced vasospasm compared to placebo (32% vs 46%, *P* < .001), but clinical outcomes were similar between the groups at 3 months.^54^

#### 3.6.3 Magnesium

Magnesium leads to vasodilatation of cerebral arteries by blocking the voltage‐dependent calcium channels. Magnesium may also block the release of glutamate, providing a potential neuroprotective benefit, and there is some suggestion of reduction in DCI associated with intravenous magnesium infusion.^55^, ^56^ Based on these properties, magnesium has been historically utilized in patients with SAH and initiated frequently on presentation. This has somewhat fallen out of standard practice given inconsistent reports on its benefits.

Several clinical studies have evaluated the role of magnesium in patients with aSAH and its effect on cerebral vasospasm and neurological outcomes. Early animal studies and phase II data reported positive results with intravenous magnesium.^56^, ^57^

The Magnesium in Aneurysmal Subarachnoid Hemorrhage (MASH) phase II trial randomized 283 patients to continuous IV magnesium infusion or placebo for 14 days after aneurysm treatment.^56^ Magnesium treatment reduced the risk of DCI by 34%, and at 3 months, the risk reduction for poor outcome was 23%. The study concluded that magnesium may reduce DCI and subsequent poor outcome.

However, there is a lack of level I evidence supporting routine use of IV magnesium for prophylaxis in patients with aSAH. In fact, several further well‐designed trials failed to show significant benefit in clinical outcomes in patients with aSAH who were treated with prophylactic magnesium infusion. The Intravenous Magnesium Sulphate for Aneurysmal Subarachnoid Hemorrhage (IMASH) trial,^58^ a randomized, double‐blinded, placebo‐controlled, multicenter phase III trial, recruited 327 patients, 169 of whom were randomized to IV magnesium sulfate. Favorable clinical outcome was observed in 64% in the magnesium sulfate group and 63% in the placebo group (OR, 1.0; 95% CI, 0.7‐1.6). The Magnesium for Aneurysmal Subarachnoid Haemorrhage (MASH‐2),^59^ another randomized phase III placebo‐controlled trial, enrolled 1204 patients, 606 receiving magnesium and 597 placebo, reported that 26.2% had poor outcome in the magnesium group compared with 25.3% in the placebo group (risk ratio [RR] 1.03, 95% CI 0.85‐1.25) and concluded that magnesium does not seem to improve clinical outcome in patients with aSAH. A meta‐analysis of seven randomized trials that included 2047 patients showed that IV magnesium does not reduce poor outcome after aSAH (RR 0.96, 95% CI 0.84‐1.10).^59^ The negative results could be related to magnesium's poor penetration in the CSF.^58^

Hypomagnesemia occurs in a large proportion of patients with SAH and could lead to worse outcomes.^60^ While maintaining magnesium within normal limits in patients with aSAH is reasonable, the current evidence does not support routine prophylactic and aggressive therapy in patients with aSAH. Furthermore, side effects associated with intravenous magnesium may offset any potential benefit (eg, hypocalcemia, hypotension).

#### 3.6.4 Milrinone

Milrinone is a phosphodiesterase 3 inhibitor. It leads to an increased level of intracellular cAMP causing vasodilation. In addition, milrinone also has potential antiinflammatory effects at the cerebral vessel wall. Milrinone's antiinflammatory effects may inhibit the abnormal proliferation of vascular smooth muscle and the remodeling process observed in patients with DCI.^61^

There have been some reports and case series showing that high‐dose IV milrinone therapy (0.1‐0.2 mg/kg intravenous bolus, then 0.75‐1.25 mcg/kg/min) for symptomatic vasospasm and DCI can lead to angiographic and neurological improvement.^62^

In the Montreal Neurological Hospital protocol study, 88 patients with aSAH received intravenous milrinone infusion with no significant side effects. About 75% of patients had a good functional outcome (modified Rankin scale ≤ 2). Systemic hypotension and tachycardia are notable side effects.

Overall, studies on milrinone have shown a safe profile and promising results, but it is still unclear to what degree it affects neurological outcome after aSAH.^63^, ^64^, ^65^ Milrinone can be administered intraarterially with good results.^65^ A study by Fraticelli et al^64^ that included 34 intraarterial infusions of milrinone reported significant improvement in angiographic vasospasm (*P*<.0001). In addition, a combination of IA and IV milrinone therapy can be attempted.^66^

Milrinone is currently used in clinical practice occasionally in cases of refractory vasospasm not responsive to other measures and utilized occasionally intraarterially in patients who undergo endovascular interventions for symptomatic vasospasm. Further prospective randomized studies are needed to prove the improved outcomes with milrinone.

#### 3.6.5 Statins

Statins are HMG‐CoA reductase inhibitors thought to have the potential to improve cerebral vasomotor reactivity by upregulating endothelial nitric oxide synthase and increasing nitric oxide biosynthesis.^67^ This can increase cerebral blood flow and could play a role in prevention and treatment of vasospasm and DCI. Additionally, statins may play a role in decreasing glutamate‐mediated excitotoxicity and help control the inflammatory response in vasospasm.^68^

There have been some positive trials evaluating the role of statin treatment in aSAH.^69^, ^70^, ^71^, ^72^

A phase II placebo‐controlled trial randomized 80 patients with aSAH to either 40 mg of daily oral pravastatin or placebo for up to 14 days. In patients treated with pravastatin, vasospasm and severe vasospasm were decreased by 32% (*P* = .006) and 42% (*P* = .044), respectively, in pravastatin‐treated patients. Pravastatin also decreased the incidence of vasospasm‐DCI by 83% (*P* < .001) and mortality by 75% (*P* = .037).^72^

However, more recent trials have not replicated those results.^68^, ^73^, ^74^ The simvastatin in aneurysmal subarachnoid haemorrhage (STASH) trial,^68^ a multicentre randomized phase 3 trial, randomly assigned 391 patients to receive simvastatin 40 mg and 412 patients to receive placebo. Favorable clinical outcome (mRS 0‐2) was not statistically different between the two groups (OR of 0.97, *P* = .803). The trial did not detect any overall benefit in the use of simvastatin and recommended against routine use of statins early in aSAH.

Given the relative safety of statins, they are still used in clinical practice (pravastatin 40 mg daily or simvastatin 80 mg daily). The latest AHA/ASA recommendation published in 2012 states that it is reasonable to administer statin therapy to patients after aSAH to prevent vasospasm despite the lack of strong evidence of benefit.^5^ If statins were to be initiated, therapy should be started early (within 48 hours) and continued for 14‐21 days. Hepatotoxicity and rhabdomyolysis are rare complications of statins but have not been prominent in most trials despite the high‐dose regimens used. More definitive trials may still be needed to confirm the effect of statins on outcomes, vasospasm, and DCI in aSAH given current mixed findings in the literature.

#### 3.6.6 Endothelin receptor antagonists

Endothelin receptor antagonists inhibit the binding of endothelin 1, a vasoconstrictive peptide that affects vascular smooth muscle contraction. Endothelin is overproduced in SAH and is linked to development of vasospasm.^75^ Clazosentan is the most commonly studied endothelin receptor antagonist in SAH.

CONSCIOUS‐1, a phase II trial that randomized 413 patients to clazosentan or placebo, reported dose‐dependent reductions in angiographic vasospasm.^76^ Three different doses were used (1, 5, or 15 mg/h). Patients who received all three dosages had a statistically significant reduction in moderate and severe angiographic vasospasm as compared to placebo, with the 15 mg/h dosage having the greatest benefit (65% relative risk reduction in angiographic vasospasm). These results led to CONSCIOUS‐2, a prospective, double‐blinded, placebo‐controlled, phase III trial that enrolled 1157 patients.^77^

A total of 728 patients received IV clazosentan (5 mg/h) and 389 patients received placebo. The group receiving clazosentan did not have statistically significant improvement in clinical outcomes.

All‐cause mortality, vasospasm‐related infarcts, DCI, and rescue therapy for vasospasm occurred less frequently in the clazosentan group (21%) compared to the placebo group (25%); however, this difference was not significant (*P* = .1).

Furthermore, this trial highlighted many side effects that included pulmonary edema, hypotension, pleural effusion, and cerebral infarction that required stopping the drug. Based on these findings, the CONSCIOUS‐3 trial that was recruiting patients was stopped early, enrolling 577 patients.^78^ Results were comparable for the 5 mg/h clazosentan dose. Clazosentan at 15 mg/h decreased the occurrence of vasospasm‐related morbidity, but did not improve functional outcomes (*P* = .266). Pulmonary complications, anemia, and hypotension were more common in patients who received clazosentan. This is also further demonstrated in meta‐analyses.^79^, ^80^

A meta‐analysis that included 2159 patients using different doses of clazosentan (1‐15 mg/h) demonstrated that high‐dose treatment (15 mg/h) is associated with reduced incidence of vasospasm and DCI at the expense of hypotension, pulmonary complications, and anemia, and no overall difference was observed in clinical outcomes.^79^ TAK‐044 is another endothelin receptor antagonist that has been studied less extensively but seems to have a similar profile to clazosentan. In one study, 207 patients were randomized to IV TAK‐044 and 213 to placebo. A trend toward decreased DCI was reported; however, there was no benefit in clinical outcomes at 3 months.^81^

Endothelin receptor antagonists may have some role in reducing vasospasm at a high dose, but reports on overall clinical benefit have been inconstant and the common side effects have pushed these medications out of standard practice.

#### 3.6.7 Tirilazad

Tirilazad functions as a free radical scavenger. This antioxidant effect is believed to confer a neuroprotective benefit in aSAH.^82^, ^83^ A trial that enrolled 1015 patients randomized to receive tirilazad at 6 mg/kg/day demonstrated significantly reduced overall mortality (*P* < .01) and improved clinical outcomes at 3 months compared to patients who received placebo (*P* < .01).^84^ The benefits of treatment with tirilazad were predominantly shown in men rather than in women.

The North American multicenter cooperative tirilazad trial studied 897 patients who received 6 mg/kg per day tirilazad or placebo.^85^ At 3 months post‐SAH, there were no significant differences in mortality, clinical outcome, or employment status. During the first 14 days after the SAH, there were no significant differences in the incidence or severity of clinical or angiographic cerebral vasospasm.

A meta‐analysis that included five double‐blind, placebo‐controlled trials involving 3821 patients found no significant difference in patients treated with tirilazad started within four days of SAH onset, compared with placebo with regard to death or poor outcome; fewer patients, however, developed DCI in the tirilazad group (OR 0.80, 95% CI 0.69‐0.93).^82^

Tirilazad studies have not proven a consistent clinical benefit following aSAH. This medication is not currently utilized in standard practice.

#### 3.6.8 Fasudil

Fasudil is a Rho‐kinase inhibitor that acts as a vasodilator. Rho‐kinase is thought to play a role in the mechanisms dictating vasoconstriction, endothelial injury, inflammation, and production of reactive oxygen species.^86^

In a trial by Shibuya et al^87^ that randomized 131 patients to receive IV fasudil for 14 days and 136 patients to placebo, fasudil reduced angiographic vasospasm by 38% from 61% in the placebo group (*P* = .0023) and symptomatic vasospasm by 30% (*P* = .0247) and significantly improved clinical outcomes according to the one‐month GOS scores (*P* = .0152). In a randomized clinical trial, the use of fasudil 30 mg as a daily IV bolus was compared with IV nimodipine.^86^ Neither the incidence of clinical vasospasm nor the occurrence of CT hypodensities differed significantly between the two groups. However, the clinical outcomes were more favorable in the fasudil group than in the nimodipine group (74.5% vs 61.7%, *P* = .040). There were no serious adverse events reported with fasudil. In this study, however, the nimodipine group received approximately half of the recommended treatment dose.

A meta‐analysis by Liu et al^88^ reported that fasudil significantly reduced the occurrence of vasospasm and DCI in SAH patients, and improved clinical outcomes.

Fasudil has been primarily studied in Japan and is currently utilized in Japan for vasospasm prophylaxis.^89^ Because of the limited number of trials, it is not as popular elsewhere. Large randomized, controlled clinical trials are needed to further establish the benefit of fasudil in aSAH.

#### 3.6.9 Cilostazol

Cilostazol is a platelet aggregation inhibitor. It may have a role in preventing microthrombus formation that is cytotoxic and may contribute to cerebral vasospasm. Cilostazol has a vasodilatory effect on the cerebral arteries as well through its inhibitory effect on phosphodiesterase 3 increasing intracellular concentrations of cAMP.^90^

In one multicenter prospective, randomized trial, 54 patients with aSAH were randomized to cilostazol treatment and 55 patients to placebo. Symptomatic vasospasm occurred in 13% of the cilostazol group vs 40% in the placebo group (*P* = .0021). The incidence of angiographic vasospasm was also significantly lower in the cilostazol group (*P* = .0055). No significant difference was found in clinical outcomes.^91^

In a meta‐analysis by Saber et al,^92^ comparing 271 patients with aSAH treated with cilostazol and 272 with placebo, cilostazol was associated with a decreased risk of symptomatic vasospasm (*P* < .001), cerebral infarction (*P* < .001), and poor outcome (*P* < .001). 

Cilostazol seems to be a safe and promising agent, and further large multicenter trials are needed before it gets widely adopted in clinical practice.

#### 3.6.10 Albumin

Human albumin is thought to have neuroprotective properties likely related to its antioxidant and antiinflammatory properties.^93^, ^94^ It has been shown to increase serum oncotic pressure and reduce cerebral edema, increase neuronal survival, and maintain blood‐brain barrier integrity.^95^, ^96^ The Albumin in Subarachnoid Hemorrhage (ALISAH) pilot study included 47 patients and identified that 1.25 g/kg/d of albumin is safe in patients with aSAH and may be associated with positive outcomes.^93^ There were no major complications. A secondary analysis showed that higher dosages of albumin were associated with a lower incidence of vasospasm, DCI, and cerebral infarction at 90 days, in a dose‐dependent fashion.^97^ Further studies are currently underway to further evaluate the role of albumin in the management of aSAH and cerebral vasospasm.

#### 3.6.11 Eicosapentaenoic acid

The sphingosylphosphorylcholine‐Rho‐kinase pathway plays an important role in vascular smooth muscle contraction. Eicosapentaenoic acid is an omega‐3 polyunsaturated fatty acid that inhibits this process and has been shown to inhibit the occurrence of cerebral vasospasm in animal models.^98^, ^99^, ^100^

A prospective multicenter randomized trial randomized 162 patients with SAH postaneurysm clipping to 900 mg of eicosapentaenoic acid three times daily for 30 days or no trial drug. The occurrence of symptomatic vasospasm (15% vs 30%, *P* = .02) and cerebral infarction vasospasm (7% vs 21%; *P* = .01) was lower in the treatment group.

Eicosapentaenoic acid is not currently used in the clinical setting, but early reports showed positive results and further studies are needed.

#### 3.6.12 Heparin and low molecular weight heparin

Heparin and low molecular weight heparin infusions have been evaluated in aSAH with inconsistent results.^101^, ^102^, ^103^ Heparin has antiinflammatory properties different than its role in anticoagulation. It is thought to attenuate the inflammatory response and restore blood‐brain barrier integrity.^104^ Wurm et al^102^ reported a significant reduction in vasospasm and DCI and improvement in 1‐month clinical outcomes in patients treated with enoxaparin vs placebo, whereas a study by Siironen et al^103^ reported no effect of enoxaparin on outcomes in aSAH and reported a slight increase in intracranial hemorrhage. The use of anticoagulation is not currently recommended routinely, outside the standard injections for prophylaxis against venous thromboembolism.

#### 3.6.13 Erythropoietin

Erythropoietin is a hormone produced primarily by the kidneys and plays an essential role in the production of red blood cells. In animal studies, erythropoietin has been shown to have some neuroprotective properties through an indeterminate mechanism that seems to be unrelated to its function in erythropoiesis.^105^, ^106^, ^107^, ^108^ It has been reported to reduce the severity of vasospasm. A phase II randomized trial that included 80 patients who received 30 000 U of intravenous erythropoietin or placebo every 48 hours for a total of 90 000 U showed a decreased incidence of severe vasospasm from 27.5% to 7.5% (*P* = .037), reduced DCI from 40% to 7.5% (*P* = .001), and better outcome at discharge (*P* = .039).^109^ In a study on seven patients with SAH and cerebral vasospasm, 30 000 IU of erythropoietin was injected for 3 days. This resulted in an increase in brain tissue oxygen tension significantly over baseline.^107^ The overall mechanisms and role in vasospasm are still unclear as well as the effect on clinical outcomes.

#### 3.6.14 Corticosteroids

The role of inflammation is central to the development of vasospasm and early brain injury post‐SAH. Corticosteroids that interfere with the glucocorticoid receptor are able to downregulate this inflammatory response. A randomized trial that administered high‐dose methylprednisolone (16 mg/kg) to patients with aSAH reported better functional outcome at one year when compared to control patients (poor outcome of 15% in the treatment group vs 34% in controls, CI 95% CI 0.5%‐37.9%).^110^ The use of corticosteroids in aSAH warrants further investigation.

#### 3.6.15 Minocycline

Matrix metalloproteinase (MMP) is strongly involved in the pathophysiology of blood‐brain barrier disruption and CNS injury.^111^, ^112^, ^113^ MMP‐9 levels are increased following SAH, and several studies have suggested a role for MMP‐9 in early brain injury after SAH and correlation between MMP‐9 levels and vasospasm in animal models.^114^, ^115^, ^116^, ^117^, ^118^, ^119^

Fischer et al^120^ evaluated blood samples in 20 patients with aSAH and showed that MMP‐9 was higher in aSAH patients compared to healthy controls (*P* < .001) and an increase in MMP correlated with occurrence of vasospasm (*P* < .05).

Minocycline is a lipophilic semisynthetic second‐generation tetracycline antibiotic that is a broad MMP inhibitor, with a good safety profile, used for other indications in humans. It has antiinflammatory effects in addition to serving as an iron chelator; thus, it has the potential of mitigating early brain injury following aSAH.^121^

Multiple trials have evaluated the neuroprotective role of minocycline in acute ischemic stroke and intracranial hemorrhage patients. A review by Malhotra et al^122^ evaluated 426 patients with ischemic or hemorrhagic stroke and reported that minocycline demonstrated a trend toward favorable functional outcome (*P* = .06) with a significant improvement in outcomes in the acute ischemic stroke subgroup (RR = 1.59, *P* = .002). Chang et al^123^ administered 10 mg/kg of minocycline over 2 hours on a daily basis for 5 days post‐ICH. They demonstrated no harmful effects and a reduction in MMP 9 levels. With minocycline's potential neuroprotective effects, positive research in stroke and intracerebral hemorrhage and with its excellent safety profile, a clinical trial in aSAH patients may be warranted.

#### 3.6.16 Deferoxamine

The amount of blood released during aSAH has been shown to be related to the degree of neurological injury and poor outcome.^124^ Abnormally high levels of iron in the brain can lead to significant oxidative damage via free radical production.^125^, ^126^, ^127^ Recent studies showed that oxidative injury and iron overload play a significant role in brain damage after intracerebral hemorrhage.^126^, ^127^ Iron deposition after intracranial hemorrhage seems to be related to oxidative injury, resulting in brain edema, neuronal cell death, and delayed brain atrophy and secondary brain injury.^128^, ^129^, ^130^

Deferoxamine is an iron chelator that chelates the unbound iron responsible for catalyzing the production of reactive oxygen species and blocks neurotoxic effects of hemoglobin.^131^ Previous studies found that deferoxamine reduces hemoglobin‐induced brain edema and decreases brain injury in experimental ICH.^130^, ^131^

Deferoxamine has currently reached clinical trials for ICH. The Deferoxamine Mesylate in Patients with Intracerebral Haemorrhage (i‐DEF) phase 2 trial^132^ evaluated clinical outcomes after deferoxamine treatment and showed promising results after 180 days (mRS 0‐2 of 45.2% in the deferoxamine group vs 35.6% in the placebo group), although the trial did not meet the primary outcomes at 90 days. Clinical outcome differences in favor of deferoxamine exceeded the prespecified futility threshold at 180 days which was not met at 90 days, showing future need to explore deferoxamine.

In a rodent SAH model study, deferoxamine treatment reduced SAH‐induced mortality (12% vs 29%, *P* < .05), brain nonheme iron concentration, iron‐handling protein expression, oxidative stress, and neuronal cell death at day 3 (*P* < .01).^125^

In another rodent study, deferoxamine injection resulted in a significant increase in hypoxia‐inducible factor (HIF)‐1 (a transcription factor that regulates the expression of various neuroprotective genes) in the brainstems of rats with aSAH. This resulted in a significant decrease in rate of cerebral vasospasm.^133^ Deferoxamine is rapidly absorbed and easily penetrates the blood barrier. Increased iron deposition after SAH and hemoglobin degradation may be a therapeutic target for patients with aSAH, and future clinical trials are needed to further establish the role of deferoxamine and iron chelators in the treatment of aSAH.

#### 3.6.17 Intrathecal and intraventricular therapy

The major advantage of delivery of treatment through an intrathecal or intraventricular route is the ability to achieve higher drug concentrations directly around the area of vasospasm and minimizing systemic toxicity.

Nicardipine is a calcium channel blocker used for treatment of hypertension, commonly utilized in its intravenous form. Intraventricular nicardipine in aSAH has been studied in small case series.^134^, ^135^ A retrospective study by Goodson et al^134^ reviewed eight patients with aSAH with refractory vasospasm who received intraventricular nicardipine 4 mg every 12 hours for a total of 5‐17 days. The study reported that seven of eight patients had moderate‐to‐good outcomes. In a case‐control study by Lu et al^135^ (14 patients who received the treatment and 14 matched aSAH patients), intraventricular nicardipine administration decreased mean flow velocity in the middle cerebral arteries, but there was no significant difference in outcomes. These studies reported minimal side effects. A phase II prospective, randomized trial evaluated the insertion of nicardipine‐releasing implants at the time of surgery. Thirty‐two patients were included. Nicardipine was more effective than placebo in decreasing the incidence of vasospasm (7% vs 73%; *P* < .05) and DCI (14% vs 47%; *P* < .05).^136^

Intraventricular nimodipine is currently being studied as well with the goal to maximize the benefit of this agent while reducing toxicity. The NEWTON trial defined 800 mg as a safe and tolerable intraventricular dose.^137^ NEWTON 2, a phase III randomized clinical trial, is currently underway.^138^

Intraventricular milrinone and sodium nitroprusside have been evaluated as well in small studies.^139^, ^140^

In addition, studies have investigated the administration of intrathecal thrombolytics in aSAH to help with the clearance of blood from the basal cisterns and ventricular system. One trial randomized 49 patients to placebo and 51 patients to intracisternal tPA at the time of aneurysm clipping. The incidence of angiographic vasospasm between the seventh and eleventh day following SAH was similar between the two groups (74.4% in the placebo group vs 64.6% in the rtPA group), with a trend toward a lower rate of vasospasm in the tPA group. Furthermore, the group that received tPA with a thick subarachnoid clot had a 56% relative risk reduction of severe vasospasm compared to placebo (*P* < .02).^141^ Hamada et al^142^ randomized 57 patients to intrathecal urokinase and 53 patients to no thrombolytics following aneurysm coiling. Patients in the treatment group experienced a significant reduction in symptomatic vasospasm (8.8% vs 30.2%, *P* < .012) and improvement in clinical outcomes at 6 months (*P* = .036) without side effects.

A meta‐analysis that included 652 patients reported positive results with an absolute risk reduction in DCI of 14.4% (*P* < .001), in poor clinical outcome of 9.5% (*P* < .01), and in death of 4.5% (*P* < .05).^143^

Intrathecal/intraventricular administration of these various agents cannot be currently recommended as level I evidence is lacking to support the routine use of locally administered therapy. Further prospective randomized trials evaluating safety and efficacy are required.

#### 3.6.18 Intraarterial treatment and angioplasty

Symptomatic cerebral vasospasm refractory to medical treatment and DCI requires further interventions. Mechanical dilation using percutaneous transluminal balloon angioplasty has become the mainstay of treatment for symptomatic focal vasospasm of the larger cerebral arteries that is refractory to medical treatment.^5^

The suggested mechanism of this approach is related to disruption of smooth muscle cells or extracellular matrix with resultant dilatation of the vessel and increased cerebral blood flow.^144^

In 1984, Zubkov et al^145^ reported the first use of transluminal balloon angioplasty for cerebral vasospasm after aSAH with good results. 

Angioplasty has high success rates in proximal arteries including the internal carotid and vertebral arteries.^146^, ^147^, ^148^ In a review by Hoh and Ogilvy, angioplasty resulted in clinical improvement in 62% of patients, significantly improved mean TCD velocities (*P* < .05), significantly improved cerebral blood flow in 85% of patients, and was associated with a 5% complication rate.^149^ One report identified that institutions that use interventional modalities such as balloon angioplasty for vasospasm had a 16% reduction in risk of in‐hospital death (RR, 0.84, *P* = .03).^150^ In clinical practice, and despite strong level I evidence, transluminal balloon angioplasty is often pursued in patients with severe vasospasm and symptoms refractory to medical treatment.

Prophylactic use of angioplasty, however, is not recommended. One multicenter randomized trial that included 170 patients with the Fisher grade III SAH randomized 85 patients to prophylactic angioplasty. The study did not show benefit in clinical outcomes from prophylactic intervention and showed a procedure‐related complication rate of 5%.^151^ Per the current AHA/ASA recommendations, balloon angioplasty before the development of angiographic vasospasm is not recommended but is reasonable in patients with symptomatic vasospasm, particularly those who are not rapidly responding to hypertensive therapy.^5^

Chemical angioplasty consisting of intraarterial administration of vasodilators is also an option that is generally used for diffuse vasospasm involving smaller arterial branches. Intraarterial nicardipine, papaverine, milrinone, nimodipine, and verapamil have been utilized in treatment of symptomatic vasospasm.^152^, ^153^, ^154^, ^155^, ^156^

Intraarterial papaverine causes vasodilatation, likely related to alteration in cAMP. It can result in transient reversal of cerebral hypoperfusion with improvement in blood flow velocities and CBF.^83^, ^157^, ^158^ There are significant limitations with its use. It has a short‐lived effect, and patients may require multiple treatments. It also has some neurotoxic effects and may result in increased intracranial pressure, brainstem depression seizures, altered mental status, and hypotension.

Given these limitations, papaverine is not routinely recommended for the treatment of vasospasm and its use is limited in aSAH.

The primary limitation of vasodilator therapy is related to the shorter duration of benefit and an increased incidence of recurrent vasospasm. Intraarterial verapamil, nicardipine, nimodipine, and milrinone appear to be safer than papaverine and provide a more durable response. Most of these agents have been tested in case series and have become widely adopted in clinical practice despite lack of strong established evidence. Randomized trials are still needed to establish safety and efficacy and to compare the different intraarterial treatments.

### 3.7 Animal models of SAH

Animal models are essential to the study of SAH and its effects. There are numerous studied animal models of SAH.^159^ Some involve the puncture or perforation of a cerebral vessel involving the use of a needle or catheter, or via a suture tied around the vessel.^160^ Endovascular approaches have also been utilized using intraluminal filaments advanced into a cerebral vessel with subsequent puncture.

Other models use injection of blood into a cistern. The cisterna magna is the most common site for blood injection in animal models. Blood may be introduced into the cisterna magna via a microcatheter or by direct puncture.^161^ Models of SAH in the anterior circulation have also been developed using injection of blood into the perichiasmatic cistern.^162^ In addition, there are single and double injection models.

Direct injection of blood is often the preferred method, given the ability of the investigator to control the initiation, volume, and rate of hemorrhage, into the cisterna magna or the prechiasmatic cistern.^163^

Originally, large animal models of SAH such as dogs and primates were used. In particular, primates were the preferred model because of similarities in their brain structure to humans. The first animal model of SAH that was developed was in primates.^164^ The basilar artery was exposed by craniotomy, and autologous blood was injected into the subarachnoid space surrounding it. However, high cost and the small number of animals that can be tested have limited this use of this model.

Canine models represent a popular model to study SAH given the large size of the animal and relatively lower cost than primates. More recently, the rat model has become one of the most utilized animal models of SAH due to low cost and ability to use large numbers of animals.^165^ Other model species studied in SAH include mice, rabbits, cats, and pigs.

Although many therapeutic agents were met with great success in preclinical animal studies, the failure in translation of these findings into clinical trials could be related to a wide variability in animal models and lack of standardized methodology. Some of the variables include volume of blood injected, the method of injection, with or without withdrawal of CSF, injection times, the use of single vs double injection models, and vascular perforation techniques. More recently, there has been an increase in acute SAH models focusing on early brain injury given the increasing clinical interest in this entity.

To note, the recently described glial‐lymphatic or glymphatic pathway, a fluid clearance pathway that subserves the flow of CSF into the brain along arterial perivascular spaces, has been found to be impaired in SAH.^166^ There appears to be impaired CSF inflow starting at 24 hours after the insult attributed to the occlusion of perivascular spaces by blood components.^167^ This could represent another target that warrants investigation in animal models of SAH.

## 4 CONCLUSION

Available treatments for aSAH continue to expand. Vasospasm and resultant DCI continue to be a significant cause of morbidity and mortality after SAH. No single‐treatment algorithm has shown to be uniformly effective in preventing post‐aSAH complications. Vasospasm after aSAH appears to be a multifactorial process, and the etiology of cerebral vasospasm remains incompletely understood.

Oral nimodipine continues to be the primary medication routinely used in practice given its neuroprotective properties and data supporting improvement in clinical outcomes. There have been many studies evaluating the role of other medical and interventional treatments for aSAH and particularly vasospasm prevention and treatment. Many of these treatments have crossed into clinical practice. There is growing evidence supporting that early brain injury in aSAH may be related to significant morbidity and mortality. This needs to be explored further. Many of the current experimental drugs may have significant roles in the treatment algorithm of aSAH, early brain injury, and vasospasm and further trials are needed.

## CONFLICT OF INTEREST

The authors declare no conflict of interest.
